# Lower Incidence Rate of Type 1 Diabetes after Receipt of the Rotavirus Vaccine in the United States, 2001–2017

**DOI:** 10.1038/s41598-019-44193-4

**Published:** 2019-06-13

**Authors:** Mary A. M. Rogers, Tanima Basu, Catherine Kim

**Affiliations:** 1 Department of Internal Medicine, University of Michigan, Ann Arbor, Michigan USA; 2 Institute of Healthcare Policy and Innovation, University of Michigan, Ann Arbor, USA

**Keywords:** Type 1 diabetes, Risk factors

## Abstract

We evaluated whether rotavirus vaccination is associated with the incidence of type 1 diabetes among children. We designed a cohort study of 1,474,535 infants in the United States from 2001–2017, using data from a nationwide health insurer. There was a 33% reduction in the risk of type 1 diabetes with completion of the rotavirus vaccine series compared to the unvaccinated (95% CI: 17%, 46%). Completion of the pentavalent vaccine series was associated with 37% lower risk of type 1 diabetes (95% CI: 22%, 50%). Partial vaccination (incompletion of the series) was not associated with the incidence of type 1 diabetes. There was a 31% reduction in hospitalizations in the 60-day period after vaccination (95% CI: 27%, 35%) compared to unvaccinated children. Overall, there was a 3.4% decrease in incidence annually in children ages 0–4 in the United States from 2006–2017 which coincides with the vaccine introduction in 2006. We conclude that rotavirus vaccination is associated with a reduced incidence of type 1 diabetes. Rotavirus vaccination may be the first practical measure that could play a role in the prevention of this disease.

## Introduction

Etiologic contributors to type 1 diabetes have garnered attention but have not yet been fully determined. Genetic predisposition plays an important role, primarily in those with HLA-DR3-DQ2 or HLA-DR4-DQ8 haplotypes^1^. But environmental factors have long been suspected as triggers of β-cell autoimmunity, including enteroviruses^2–4^. Recently, a report from Australia indicated that the incidence rate of type 1 diabetes decreased after the introduction of the oral rotavirus vaccine^5^. There appeared to be a cohort effect, with a 14% reduction in rates in children ages 0–4 years of age but not in older children^5^. Additionally, the SEARCH for Diabetes in Youth Registry observed a 1.5% annual decrease in the incidence of type 1 diabetes in children 0–4 years from 2002 to 2012 which coincides with the introduction of the rotavirus vaccine in the United States in 2006^6^.

Two types of the rotavirus vaccine are routinely used in the United States: pentavalent RotaTeq introduced in 2006 and given in 3 doses at 2, 4 and 6 months; and monovalent Rotarix introduced in 2008 and given in 2 doses at 2 months and 4 months^7^. Because nationwide data regarding dates, types, and doses of vaccines are available from health insurers, we conducted a nationwide study to investigate the hypothesis that the rotavirus vaccine may reduce the likelihood of type 1 diabetes in children.

## Results

### Study participants

**Figure 1:**
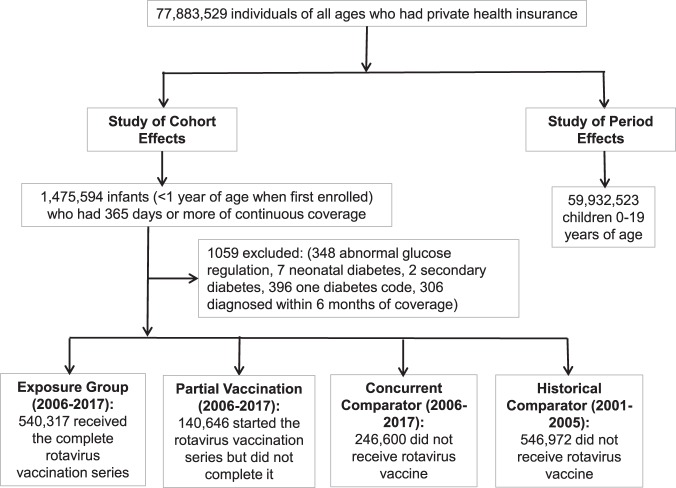
Flow Diagram of Study Participants.

There were 77,883,529 individuals of all ages with private health insurance during 2001–2017 in this database. There were 1,475,594 infants when first enrolled with continuous coverage for at least 1 year (Fig. 1). Children with evidence of diabetes before completion of the vaccination series or within the first 6 months of coverage (for those who did not receive the vaccine) were removed. These were infants with abnormal glucose regulation (n = 348), neonatal diabetes (n = 7), diabetes secondary to other conditions (n = 2), only one diabetes diagnosis code (n = 396), and diabetes codes but within the first 6 months of coverage (n = 306, of whom 250 were diagnosed when hospitalized). The remaining 1,474,535 infants formed our study cohort. In the entire cohort, the time under observation ranged from 1.0 to 16.5 years, with a median of 3 years (IQR 1.75, 5.17).

### Development of diabetes

The mean number of diabetes diagnosis codes (per patient) was 32 (SD 39) and median number was 19 (IQR: 4, 47). All had received anti-diabetic medications. The mean age at diabetes diagnosis was 3.5 years in the vaccinated group, 3.0 years in the partially vaccinated group, and 3.8 years in the concurrent unvaccinated group (p = 0.0499) (Table 1). Children in the historical comparison group were followed for a longer period of time and their mean age at first diagnosis was 6.6 years.

**Table 1:** Study Participants Stratified by Vaccination Status.

Group	Number of Children	Years of Follow-up, mean (SD)	Years of Follow-up, median (IQR)	Sum Years of Follow-up	Number who developed diabetes
All					
Completed vaccination series, 2006–2017	540,317	2.91 (2.16)	2.28 (1.20, 4.08)	1,573,539	192
Partially vaccinated, 2006–2017	140,646	2.81 (2.21)	2.08 (1.16, 3.91)	395,443	81
Not vaccinated, 2006–2017	246,600	3.26 (2.63)	2.41 (1.17, 4.59)	805,042	166
Not vaccinated, 2001–2005	546,972	4.12 (3.54)	2.91 (1.42, 5.75)	2,252,514	834
Girls					
Completed vaccination series, 2006–2017	263,528	2.92 (2.16)	2.28 (1.20, 4.09)	768,237	95
Partially vaccinated, 2006–2017	68,448	2.81 (2.22)	2.08 (1.16, 3.92)	192,475	33
Not vaccinated, 2006–2017	119,840	3.28 (2.64)	2.42 (1.17, 4.67)	392,756	83
Not vaccinated, 2001–2005	265,458	4.12 (3.54)	2.91 (1.42, 5.75)	1,094,839	395
Boys					
Completed vaccination series, 2006–2017	276,730	2.91 (2.16)	2.28 (1.20, 4.08)	805,182	97
Partially vaccinated, 2006–2017	72,187	2.81 (2.21)	2.08 (1.16, 3.91)	202,952	48
Not vaccinated, 2006–2017	126,718	3.25 (2.62)	2.34 (1.17, 4.59)	412,218	83
Not vaccinated, 2001–2005	281,396	4.11 (3.53)	2.91 (1.42, 5.75)	1,157,428	439

In the children who received the complete rotavirus vaccination series, there were 192 children who developed diabetes. The incidence rate of type 1 diabetes was 12.2/100,000 person-years (Table 2). In girls, the incidence rate was 12.4/100,000 person-years and, for boys, was 12.0/100,000 person-years. In the children who were partially vaccinated, 81 developed diabetes during the follow-up period, for an incidence rate of 20.5/100,000 person-years. In the unvaccinated group (concurrent comparator, followed from 2006–2017), there were 166 children who developed diabetes, for an incidence rate of 20.6/100,000 person-years.

**Table 2:** Incidence Rate Ratio and Difference for Type 1 Diabetes, comparing Rotavirus Vaccination with No Rotavirus Vaccination, 2006–2017.

Category	Incidence Rate*	Incidence Rate Ratio	95% CI	Incidence Rate Difference*	95% CI
All					
Completed Vaccination Series	12.2	0.59	0.48, 0.73	−8	−12, −5
Partially Vaccinated	20.5	0.99	0.75, 1.30	0	−6, 5
Not vaccinated	20.6	1.00	(reference)		
Sex					
Girls					
Completed Vaccination Series	12.4	0.59	0.43, 0.80	−9	−14, −4
Partially Vaccinated	17.1	0.81	0.52, 1.23	−4	−1, 3
Not vaccinated	21.1	1.00	(reference)		
Boys					
Completed Vaccination Series	12.0	0.60	0.44, 0.81	−8	−13, −3
Partially Vaccinated	23.6	1.17	0.81, 1.70	4	−4, 11
Not vaccinated	20.1	1.00	(reference)		
Year of Birth					
2006–2011					
Completed Vaccination Series	14.0	0.67	0.53, 0.84	−7	−11, −3
Partially Vaccinated	21.9	1.04	0.76, 1.41	0	−5, 7
Not vaccinated	21.0		(reference)		
2012–2016					
Completed Vaccination Series	8.2	0.46	0.26, 0.86	−10	−18, −1
Partially Vaccinated	14.2	0.80	0.38, 1.69	−3	−14, 7
Not vaccinated	17.7		(reference)		

*Per 100,000 person-years.

There was a 41% reduction (95% CI: 27%, 52%) in the incidence of type 1 diabetes in children who received the entire rotavirus vaccination series compared to children who did not receive the vaccine during the same time period. There was no reduction in the incidence of type 1 diabetes for children who were only partially vaccinated (IRR 0.99; 95% CI: 0.75, 1.30; p = 0.967).

We also stratified by year of birth. There was a 33% reduction in the risk of type 1 diabetes after receiving the complete vaccination series compared to no vaccination in those born in 2006–2011. For the infants born in 2012–2016, there was a 54% reduction in risk.

**Figure 2:**
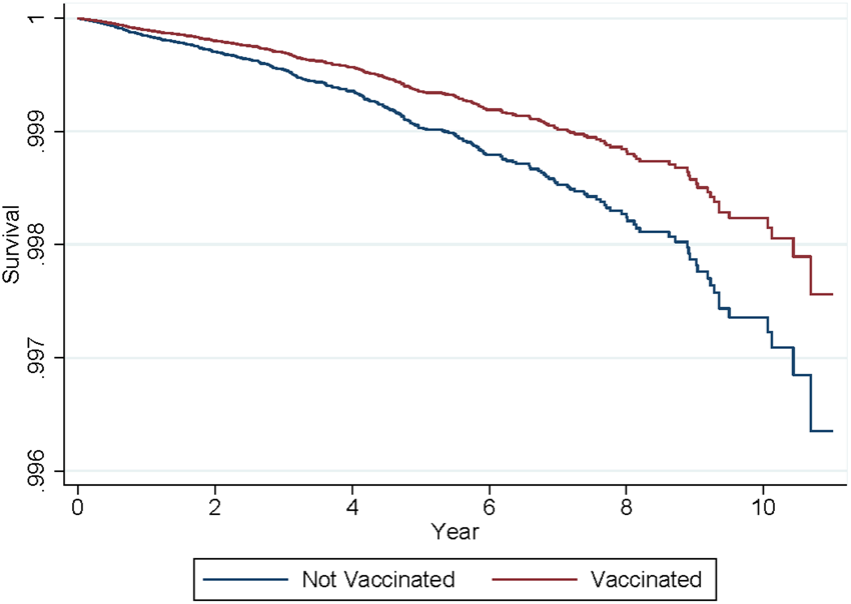
Cox Proportional Hazards Survival Curve for the Incidence of Type 1 Diabetes Comparing Infants who Completed the Rotavirus Vaccination Series and Infants who were not Vaccinated for Rotavirus, 2006–2017.

Using Cox proportional hazards regression, we compared infants who received the entire rotavirus series to those who were unvaccinated in the concurrent cohorts (2006–2017). The unadjusted hazard ratio (HR) was 0.64 (Table 3). The adjusted HR was 0.67 (95% CI: 0.54, 0.83) indicating a 33% reduction in the risk of type 1 diabetes. The underlying assumption of proportional hazards was met (p = 0.369). When year of birth was added to the model, the HR was 0.70 (95% CI: 0.56, 0.88). Survival curves are shown in Fig. 2.

**Table 3:** Hazard Ratio for the Association between Rotavirus Vaccination and Type 1 Diabetes, 2006–2017.

	Unadjusted Hazard Ratio	95% CI	p value	Adjusted Hazard Ratio	95% CI	p value
Vaccination						
Completed Rotavirus Series	0.64	0.52, 0.79	<0.001	0.67	0.54, 0.83	<0.001
Not vaccinated				1.00	(reference)	
Sex						
Girls				1.04	0.84, 1.28	0.734
Boys				1.00	(reference)	
Season of Birth						
Spring				1.55	1.12, 2.14	0.008
Summer				1.36	0.98, 1.90	0.068
Autumn				1.42	1.02, 1.97	0.039
Winter				1.00	(reference)	
Residence at Birth						
New England, New Jersey				1.00	(reference)	
New York, Pennsylvania				0.84	0.49, 1.44	0.533
Middle Atlantic				0.78	0.49, 1.26	0.315
Southeast				0.64	0.41, 1.00	0.051
Michigan, Indiana, Ohio, Kentucky				0.81	0.50, 1.32	0.399
North Central				0.55	0.33, 0.92	0.023
Middle Central				0.58	0.34, 0.99	0.047
South Central				0.51	0.32, 0.82	0.005
Mountain				0.90	0.57, 1.42	0.640
Pacific				0.87	0.56, 1.35	0.542

Sensitivity analyses indicated a significant association between rotavirus vaccination and the use of insulin (HR = 0.71), hospitalization for type 1 diabetes (HR = 0.70), and for two or more type 1 diabetes codes with the use of insulin (HR = 0.70) (Table 4).

**Table 4:** Sensitivity Analysis for the Association between Rotavirus Vaccination and Type 1 Diabetes, 2006–2017, using Different Methods of Detection.

Definitions	Unadjusted Hazard Ratio*	95% CI	p value	Adjusted Hazard Ratio**	95% CI	p value
Use of insulin	0.73	0.56, 0.95	0.017	0.71	0.54, 0.94	0.015
Hospitalization for type 1 diabetes	0.68	0.51, 0.92	0.013	0.70	0.50, 0.97	0.031
Two or more type 1 diabetes codes and the use of insulin	0.73	0.56, 0.95	0.019	0.70	0.53, 0.93	0.014

*Completed the entire rotavirus vaccination series versus no rotavirus vaccination.

**Adjusted for sex, season of birth, birth year, and region of the United States.

There was no association between sex and the incidence of type 1 diabetes in this cohort. Infants born in the winter were less likely to develop type 1 diabetes than those in the spring or autumn. Infants who resided in the New England states and New Jersey were significantly more likely to develop type 1 diabetes than infants in the central sections of the country.

### Type and cost of the rotavirus vaccine

Of the 540,317 infants who received the complete vaccination series, 83.3% (n = 450,319) received the pentavalent RotaTeq. When regressed simultaneously in the survival analysis, the hazard ratio (HR) for infants who received the entire RotaTeq series was 0.63 (95% CI: 0.50, 0.78) and the HR for completion of the Rotarix series was 0.73 (95% CI: 0.50, 1.07). When modeled separately, the HR for pentavalent RotaTeq was 0.66 (95% CI: 0.53, 0.81) and for the monovalent Rotarix was 0.90 (95% CI: 0.62, 1.31).

In 99.6% of instances in which the rotavirus vaccine was given, there was $0.0 copayment for the patient.

### Comparison with historical cohort

We also investigated incidence rates in the historical cohort. Because the observation period was longer in the historical unvaccinated cohort (born 2001–2005 and followed through 2017), we compared these children with those receiving the complete vaccination series by right-truncating all follow-up at 5.0 years in both of these groups. During this 5-year period, the incidence rate of type 1 diabetes was 9.3/100,000 person-years in the vaccinated group and 20.5/100,000 person-years in the unvaccinated group. The IRR was 0.45 (95% CI: 0.37, 0.56), indicating a 55% decreased rate in the children who received the entire vaccination series compared to those who were not vaccinated.

### Specificity of vaccination

Because infants received multiple vaccinations on a similar schedule, we wished to evaluate whether it was specifically the rotavirus vaccine (rather than the other vaccines) that was associated with the onset of type 1 diabetes. It was not possible to disentangle these effects in the concurrent cohort from 2006–2017 because the infants received the vaccinations on the same day. Therefore, we used the historical cohort born from 2001–2005 and selected the infants who received their first three diphtheria, tetanus, and pertussis (DTaP) vaccines (2, 4, 6 months). These infants (vaccinated for DTaP but not vaccinated for rotavirus) were compared with the infants who completed their vaccination series from 2006–2017 (who received both rotavirus and DTaP vaccines). Because the observation time differed in these two cohorts, we right-truncated their follow-up at 5 years for both groups. The hazard ratio was 0.44 (95% CI: 0.36, 0.54) indicating that children who completed the rotavirus vaccination series (and DTaP series) were 56% less likely to develop type 1 diabetes than children who only received the DTaP series. When adjusted for sex, season of birth and region of the country, the hazard ratio remained 0.44 (95% CI: 0.36, 0.54).

### Hospitalizations after vaccination

In the 60-day window after vaccination, there were 2954 hospitalizations in the 540,317 children who completed the entire rotavirus series. In the 60-day window for the concurrent comparator (unvaccinated, n = 246,600), there were 1944 hospitalizations. The IRR for hospitalizations was 0.69 (95% CI: 0.65, 0.73; p < 0.001). That is, there was a 31% reduction in hospitalizations for children who were vaccinated compared to children who were not vaccinated in this 60-day period.

Hospitalizations for enteritis due to rotavirus were reduced by 93.9% after completing the vaccination series. The IRR comparing vaccinated to unvaccinated was 0.061 (95% CI: 0.028, 0.118; p < 0.001) during the 60-day period after vaccination. There were 10 such hospitalizations in children who were vaccinated and 75 such hospitalizations in unvaccinated children.

### Variation in vaccination rates

We examined the percentage of children receiving the entire vaccination series, as well as the percentage receiving a partial series from 2006–2017 (Table 5). When grouped by the first digit of the residential zip code, the percentage of infants receiving the complete rotavirus vaccination series ranged from 48.9% in New England and New Jersey to 63.8% in the middle central states (Illinois, Missouri, Nebraska, Kansas). The percentage of infants not receiving the rotavirus vaccine (even the first dose) ranged from 22.5% to 34.8%. The areas with the greatest rates of unvaccinated infants were New England states and the Pacific.

**Table 5:** Percentage of Infants Receiving Rotavirus Vaccination by Area, 2006–2017.

First Digit of Zip Code	Description	Number Completed Vaccination Series	Number Partially Vaccinated	Number Not Vaccinated	Total	Percent Completed Vaccination Series	Percent Partially Vaccinated	Percent Not Vaccinated
0	New England, New Jersey	28,568	9,538	20,365	58,471	48.9%	16.3%	34.8%
1	New York, Pennsylvania	28,427	7,307	14,461	50,195	56.6%	14.6%	28.8%
2	Middle Atlantic	50,843	12,224	21,072	84,139	60.4%	14.5%	25.0%
3	Southeast	84,721	21,249	35,492	141,462	59.9%	15.0%	25.1%
4	Michigan, Indiana, Ohio, Kentucky	45,599	11,291	19,451	76,341	59.7%	14.8%	25.5%
5	North Central	56,454	11,536	21,362	89,352	63.2%	12.9%	23.9%
6	Middle Central	50,302	10,768	17,749	78,819	63.8%	13.7%	22.5%
7	South Central	86,854	22,483	33,411	142,748	60.8%	15.8%	23.4%
8	Mountain	50,229	13,322	24,480	88,031	57.1%	15.1%	27.8%
9	Pacific	58,320	20,928	38,757	118,005	49.4%	17.7%	32.8%

### Incidence rates by calendar year

**Figure 3:**
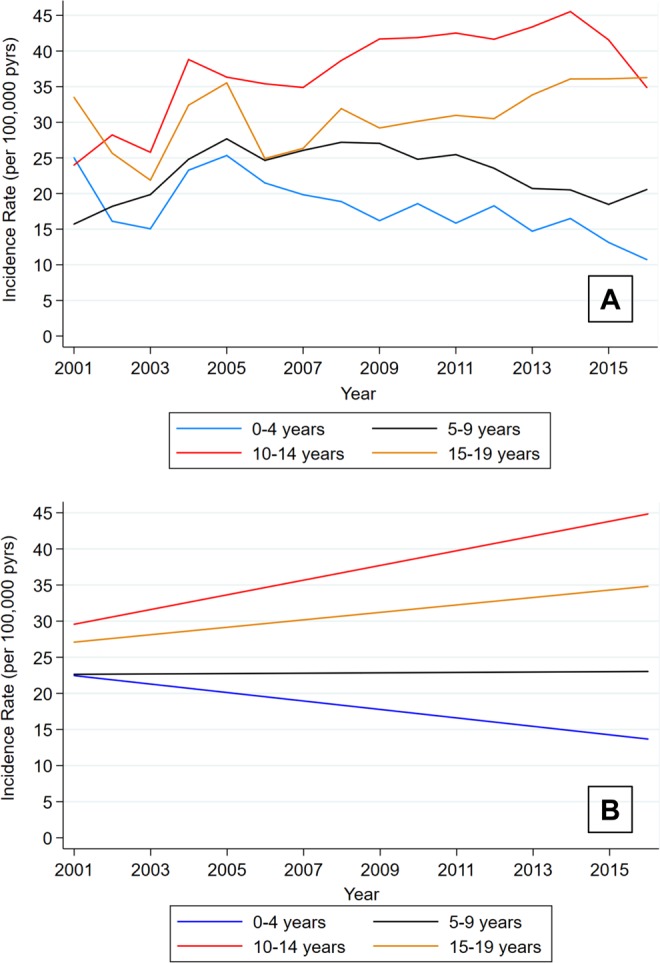
Incidence Rates of Type 1 Diabetes in the United States, Ages 0–19, Years 2001–2016 (**A**: Rates and **B**: Linear Fit).

Incidence rates of type 1 diabetes for ages 0–19 years in the United States (n = 59,932,523 children and adolescents with two type 1 diabetes diagnosis codes with the use of insulin) were graphed, with stratification by age group (Fig. 3). There was a 3.4% decrease in the rates (95% CI: 1.6%, 5.1%; p < 0.001) annually in children ages 0–4. There was no significant change in the rates for children ages 5–9 (IRR 1.00; 95% CI: 0.98, 1.02; p = 0.867). For children ages 10–14, there was a 3.0% increase in rates annually (95% CI: 1.6%, 4.4%; p < 0.001). There was a 1.6% increase in annual rates (95% CI: 0.3%, 2.9%) in youth ages 15–19 (p = 0.013). The decrease in rates over time for children ages 0–4 was significantly different than the other slopes (p = 0.002 ages 5–9; p < 0.001 ages 10–14; p < 0.001 ages 15–19 for the interaction term).

Using all children combined, there was a 7.2% annual increase in the incidence prior to 2006 (95% CI: 3.4%, 11.2%) and a 6.9% decrease afterward (95% CI: 2.8%, 19.9%). The pre-to-post change in slope was significant (p = 0.007).

## Discussion

We found a significant reduction in the incidence of type 1 diabetes in children who received the entire rotavirus vaccination series compared to both a contemporary cohort and a historical cohort who were not vaccinated. There was no reduction in incidence in children who received only part of the recommended vaccination series. The pentavalent vaccine, in particular, was associated with lower risk. These results support the findings from Australia^5^ and the decrease in type 1 diabetes incidence in children <5 years observed in the SEARCH for diabetes in youth registry^6^. The rotavirus vaccine appeared safe with fewer hospitalizations among those vaccinated.

Evidence linking rotavirus with type 1 diabetes stems from both animal^3,8^ and human studies^2,9,10^. Rotavirus infection has been shown to accelerate the destruction of β cells in diabetes prone mice^3^ and lead to pathogenic infection of the pancreas^8^. Honeyman and colleagues assembled a cohort of 360 high-risk children and found that islet antibody levels significantly increased with repeated rotavirus infections^2^. A recent study in children found that greater viral load of enteroviruses in the gut was associated with islet autoimmunity, a feature of type 1 diabetes^9^. In a case-control study of enterovirus RNA in the blood, RNA positivity peaked in the 6 month time period before the first autoantibodies were detected in children with type 1 diabetes^10^. Our findings build upon these prior results. Our data suggested that the pentavalent vaccine may be more likely to reduce the risk of type 1 diabetes than the monovalent vaccine. However, it is important to note that there were children who received the entire rotavirus vaccination series but still developed type 1 diabetes. Therefore, there may be other concomitant factors involved in the pathogenesis of the disease.

In our study, the vast majority of the infants who received the rotavirus vaccine also received the vaccine for diphtheria, tetanus, and pertussis on the same days. This is in accordance with recommended guidelines^7^. Children who received all the vaccines on the same day (with rotavirus vaccine) were less likely to develop type 1 diabetes. Therefore, concerns regarding the safety of administering multiple vaccines at the same time should be assuaged. These children, as a group, experienced a benefit.

Because the rotavirus vaccine is given to birth cohorts, it will take years before the entire population of children in the United States is vaccinated for rotavirus. Although the vaccine was introduced nationwide in 2006, only a small fraction of the population received it at that time. The first birth cohort receiving the vaccine included infants who completed the series in 2007. Each year since, a new birth cohort received the vaccine. Today, there remains a large proportion of children (most born before 2006) who did not receive the vaccine. While we are witnessing the beginning of an observed trend, it should be monitored to assess whether these patterns continue.

We found that a relatively large proportion of infants in the study did not receive the complete vaccination series. Some of this may be due to natural lags in adoption after the introduction of a new vaccine. This may also be a consequence of parental reticence to vaccinate; parental attitudes and knowledge have been identified as major contributors to non-vaccination throughout the world^11^. In our study, New England states had lower vaccination rates and higher rates of type 1 diabetes. Pacific states also had lower rates of vaccination. The CDC shows variation in rotavirus vaccination across the states, with national coverage ranging from approximately 70–75% in 2013–2017^12^. Cost does not appear to be a major impediment because, in our study, there was no copayment 99.6% of the time the vaccine was administered. In addition, the rotavirus vaccine is given orally so the reticence to vaccinate due to fear of needles, common in children and young mothers, should not be an issue^13^. This speaks to finding other avenues to impact vaccination coverage; multicomponent approaches that are dialogue-based have shown some success^14^.

Our study has limitations; it is observational and not experimental. However, it would be unethical to conduct a trial whereby children were deliberately not given the rotavirus vaccine to observe the incidence of diabetes because the rotavirus vaccine has been shown to prevent death and hospitalizations from gastroenteritis^15^. In addition, there is the possibility of incomplete information either on vaccination status or diabetes diagnosis. Because there may be differences in people who are vaccinated versus those who are not, potential confounding factors may be responsible for these findings. One way we addressed this was by comparing vaccinated infants with other vaccinated infants. Children receiving the rotavirus and DTaP vaccines were less likely to develop type 1 diabetes than children vaccinated with just the DTaP vaccine. This provides evidence that it is the rotavirus vaccine, itself, that may be involved in the etiology of type 1 diabetes. Another limitation is that we cannot discern whether the rotavirus vaccine is associated with a lower lifetime risk of developing type 1 diabetes or whether it merely delays the onset of the disease. Longer longitudinal studies are necessary for evaluation.

We conclude that receiving the rotavirus vaccine is associated with a lower risk of developing type 1 diabetes in children. Type 1 diabetes is a serious lifelong disease with considerable impacts on one’s quality of life, requiring daily intensive management. While additional studies are needed to explore this association in more detail, it is possible that rotavirus vaccination may be the first practical measure that could play a role in the prevention of this disease.

## Methods

We designed a cohort study using data from a nationwide health insurer in the United States (Clinformatics DataMart^®^ Database; available through OptumInsight, Eden Prairie, Minnesota, USA) from January 1, 2001 to June 30, 2017. The study was reviewed by the institutional review board at the University of Michigan and was deemed exempt. The study was carried out in accordance with National of Institutes of Health guidelines for studies that utilize de-identified data from humans.

In this longitudinal design, the eligibility requirements were: (a) infant (<1 year of age) at the start of insurance coverage, and (b) continuous health insurance coverage for a minimum of 365 days. Infants were categorized as receiving the entire rotavirus series (3 doses of RotaTeq or 2 doses of Rotarix), partial vaccination (at least one dose without completion of the series), and unvaccinated infants. Unvaccinated infants were further characterized in 2 ways: infants not vaccinated for rotavirus during years 2006–2017 and infants who were born earlier (2001–2005), prior to the introduction of the vaccine. All groups were observed until 2017 or when insurance coverage ended. For the vaccinated, observation time started with the last rotavirus vaccination date. For unvaccinated, the starting time of observation began at 6 months (182 days) from the first eligible date.

Vaccination status was determined through Current Procedural Terminology codes (90680, 90681) which were available in the medical and facility files. Diabetes was determined through ICD-9 and ICD-10 diagnosis codes which were available in the medical and confinement files. An incident diabetes case was determined by, at minimum, two diabetes diagnoses from inpatient and outpatient files.

Preliminary analyses included calculation of incidence rates of type 1 diabetes by vaccination status. Incidence rate ratios (IRR) and incidence rate differences (IRD) were determined with 95% confidence intervals (CI). Results were stratified by sex and year of birth. Alpha was set at 0.05, 2-tailed.

Cox proportional hazards regression was used to compare time to diabetes diagnosis in children who received the complete rotavirus vaccination series to children who were unvaccinated for rotavirus. We adjusted for sex, season of birth, and region of the country (identified through the first digit of residential zip code) to account for the clustering of the population by ancestry.

In secondary analyses of vaccine safety, we calculated hospital admission rates in the 60-day window after receipt of the vaccine. For those children who were not vaccinated, we utilized the 60-day window after the first 6 months of insurance coverage (enrollment date +182 days for the start of the window).

To evaluate whether incidence rates changed over calendar time, we utilized all children and adolescents in the database (ages 0–19, years 2001–2017) similar to previous research^16^. Negative binomial regression was used to model rates over time, offset by person-years. A piecewise model was used with a knot at year 2006 to assess pre-post changes in slope.

The data are available through OptumInsight, Eden Prairie, Minnesota, USA.
